# The First Paragraph of *the Organon*

**Published:** 1892-03

**Authors:** 


### HOMŒOPATHIC PHYSICIAN,

A MONTHLY JOURNAL OF

HOMŒOPATHIC MATERIA MEDICA AND CLINICAL MEDICINE.

"If our school ever gives up the strict inductive method of Hahnemann, we are lost, and deserve only to be mentioned as a caricature in the history of medicine."—CONSTANTINE HERING.

Vol. XII. MARCH, 1892. No. 3.

### EDITORIAL.

THE FIRST PARAGRAPH OF THE *Organon* reads thus: "The first and *sole* duty of the physician is to restore health to the sick. This is the true art of healing."

At first sight this would seem to be a surprising and superfluous declaration to make, since the whole idea of medicine is the cure of the sick.

When, however, one has really gotten into the current of thought of the medical men of to-day and examined their processes, it is apparent that there is great need of the first paragraph of the *Organon* to recall physicians to their actual duty.

Having made of disease an entity, and having built up elaborate theories of pathological action of the tissues, and having withal based prescriptions upon these theories, they have met with so many failures in their efforts to cure that they have despaired of ever bringing therapeutic measures to a condition of efficiency and reliability. Skepticism of the value of *any* curative measures has slowly and surely taken possession of the best minds in the profession, and they have, in consequence, turned their attention to an investigation of the causes of disease and of the nature of the pathological processes involved. They have pushed these investigations to a great length with amazing perseverance, industry, and ingenuity. The most minute and painstaking examinations of every perverted action of the system and morbid change in the tissues have been made in the intense desire to solve the problems afforded by the various phases of disease. So absorbed have these investigators become in their keen quest that they have lost sight of the prime object of all medical learning, the relief of the patient. They seem to think that their whole duty to the patient is done if they " find out what was the matter." Indeed a physician has been heard to exclaim triumphantly, after the death of his patient: " Well, I found out what was the matter, and that's the principal thing after all."

This close examination of minute changes in the tissues has caused more or less forgetfulness of the contemplation of all the phenomena which go to make up the case as a whole. They forget to consider the case as a " sick individual." Consequently their measures are directed to individual symptoms, and to theoretical considerations of the effect of drugs upon particular pathological processes.

Thus they become, on the one hand, symptom coverers, and on the other pathological theorists. Meanwhile for want of a perspective view of the case as a *sick man*, the patient languishes and may die. It is no wonder, therefore, that Hahnemann opens his argument for Homœopathy with the proposition " the first and *sole* duty of the physician is to restore health to the sick."

Whoever faithfully applies the homœopathic law, with the single remedy, and minimum dose, is at once placed in a position to carry out this first proposition of Hahnemann. His obedience to the law of similars will lead him to examine the patient as a whole; taking account of every symptom, ignoring none, attributing none to imagination because not explainable by the pathological knowledge of the day. No loss of time in the helping of the patient occurs because of elaborate microscopical examinations. The time that might be expended upon these investigations is used in selecting and applying the true simillimum, and thus he arrives at " the perfection of a cure " which, as stated in paragraph second, " consists in restoring health in a prompt, mild, and permanent manner."

